# Developing a Comprehensive Evidence-Based Service Package for Toddlers with Autism in a Low Resource Setting: Early Detection, Early Intervention, and Care Coordination

**Published:** 2019-04

**Authors:** Hadi Zarafshan, Mohammad Reza Mohammadi, Farid Abolhassani, Seyed Abbas Motevalian, Vandad Sharifi

**Affiliations:** 1 Psychiatry and Psychology Research Center, Tehran University of Medical Sciences, Tehran, Iran.; 2 National Institute of Health Research, Tehran University of Medical Sciences, Tehran, Iran.; 3 Department of Epidemiology, School of Public Health, Iran University of Medical Sciences, Tehran, Iran.; 4 Department of Psychiatry, Tehran University of Medical Sciences, Tehran, Iran.

**Keywords:** *Autism*, *Care Coordination*, *Early Detection*, *Early Intervention*

## Abstract

**Objective:** The number of children with autism, who have many unmet needs, is increasing dramatically. However, the existing evidence shows that early identification and intervention are effective in reducing the later costs and burdens of autism spectrum disorder (ASD). Thus, the present study aimed to develop evidence-based services for children with autism in Iran to reduce its impacts on the affected children and their families and to decrease its burden on the society.

**Method** **:** A 3-step study was conducted based on a modification of the Replicating Effective Programs (REP) framework (step 1: need assessment and situation analysis; step 2: identifying current evidence-based services; step 3: designing the first draft of the package and its core elements). Each step was conducted by a specific methodology.

**Results: **By considering the obtained data, it was found that a package of services with 4 core components to respond to the perceived needs in Iran was needed: (1) early detection of at-risk children; (2) care coordination and facilitation of access to current services; (3) implementation of an evidence-based early intervention program; and (4) training interventionists using an effective educational framework based on evidence-based material.

**Conclusion: **REP framework was used in the present study, which has been shown to be effective in adapting and implementing health care services. By considering the preconditions of REP, a comprehensive package of services, with 4 components was designed for toddlers with autism in Iran. The next step will be to study this package using a multicenter hybrid effectiveness-implementation randomized control trial.

Autism spectrum disorder (ASD), as the most severe neurodevelopmental disorder, is characterized by restricted patterns of behavior and interests and significant impairments in social communication (1). The worldwide prevalence of ASD has increased in recent years. Based on a recent study, prevalence of autism in the United States has increased from 0.67% in 2000 to 1.47% in 2010 and 2.58% in 2016 (2).

Autism usually affects different domains of abilities during patients’ lifetime; and its complexity and demands lead to significant burdens on families and societies (3). 

The lifelong costs of ASD have been reported as 2.4 and 2.2 billion dollars in the United States and United Kingdom, respectively (4). However, it is shown that early identification and intervention can reduce symptoms of autism and its negative effects and its long-term costs (5-7). Early intensive behavioral interventions (EIBI) have reduced the lifetime costs of autism about 1 103 067 Euros per individual in the Netherlands (7). 

Also, another new study has shown that early intervention based on the Early Start Denver Model (ESDM) decreases the need for occupational therapy, speech therapy, and special education and leads to annual savings of about $19 000 per child with autism (8).

According to the recent world bank classification, Iran is a upper-middle income country located in the Middle East, and epidemiologic studies have shown the considerable prevalence of autism in this country. In a school-based study conducted in Shiraz, a large city located in the center of Iran, 1.9% and 0.5% of school-aged children were suspicious of having autism and Asperger disorders, respectively (9). A secondary analysis on more than 1 000 000 records obtained from the routine screening of 5-year old children, conducted by the “Special Education Organization”, revealed a prevalence of classic autism to be 6.26 per 10 000 children (10). According to the Diagnostic and Statistical Manual of Mental Disorders-Fifth Edition (DSM-5), the worldwide prevalence of autism spectrum disorder (ASD) is estimated to be about 1% (1). If this conservative prevalence rate and also the results of the last national census is taken into account, it could be estimated that more than 70 000 children younger than 5 years, 135 000 younger than 10 years, and almost 200 000 younger than 15 years are at risk of autism in Iran, and, unfortunately, many of them remain unrecognized. In Iran, similar to any other country, ASD has negative impacts on families and the society (11).

No rigorous evidence has been published on the burden and costs of ASD in Iran. However, some studies on Iranian families with a child with autism have shown that they spend much of their income on the education and care of the child with autism and they usually do not have enough time and resources to respond to the needs of other members of the family (11, 12). Moreover, these families expressed that they do not have access to appropriate services and supports (12).

Given the high number of children with autism in Iran and their unmet needs and considering the existing evidence on the effectiveness of early identification and intervention in reducing the later costs and burdens of ASD, it seems reasonable to plan and implement evidence-based services for people with autism.

The aim of this study was to develop an evidence-based service package for children with autism in Iran to reduce the impacts of this disorder on affected children and their families and to lower its burden on the society. Thus, a modification of the Replicating Effective Programs (REP) framework, recommended by the U.S. Center for Disease Control and Prevention (CDC), was used to plan for effective health services in community-based settings (13).

## Materials and Methods

In this study, a modified version of the REP framework, shown to be effectively used in packaging health services, including mental health services, was used (14, 15). The REP has 4 phases: 1) preconditions, 2) preimplementation, 3) implementation, and 4) maintenance and evaluation (13). This study included the results of the study which was designed based on the components of phase one of the REP framework. The aim of the present study was to draft the service package. To achieve this goal, a 3-step study (need assessment and situation analysis; searching for existing evidence-based services; designing the first draft of the package and its core elements) was done. See Table 1 for details of REP and its correspondences.

Before starting the project, an expert panel were selected to control the study process, make decisions about any adjustments, summarize the results, and determine the components of the service package. The panel consisted of 1) a professor of child psychiatry who is the head of a research center in the field of psychiatry and mental health. Also, he had been previously in charge of the Social Welfare Organization of Iran; 2) a general psychiatrist who is expert in mental health service development and implementation and in community-based and collaborative models of care; 3) a specialist (MD) in the field of health care service development who is the head of Department of Health Services, National Institute of Health Research; 4) an epidemiologist (MD, PhD) with experience in the field of mental health research; and 5) a psychologist who has experience in working with young children with autism and their families. 

**Step one**

Need assessment and situation analysis

In this study, to identify the current condition of service delivery to patients with autism and their families, three studies were conducted:

***Study one***

A systematic review was done on studies in the field of autism by Iranian researchers on local samples.

The aim of this study was to obtain a clear picture of scientific works about autism in Iran and presence of any evidence about epidemiology, service development, and infrastructures to service implementation. Also, seven databases, including three Persian (Magiran, SID, Irandoc) and four English language databases ((PubMed, Scopus, ProQuest, PsycInfo), were systematically searched to retrieve all published articles in the field of autism by Iranian researchers. All searches were conducted from 1979 to 2015.

The bibliometric information of all obtained papers was extracted and categorized based on the “subject area” into 7 areas adopted from the criteria used in Pellicano et al (16). The result of this study has been published as a scientometric study entitled, “Autism Research in Iran: A Scientometric Study” (17).

***Study two***

A qualitative study was conducted using a semi-structured interview with a sample of parents of children with autism.

The full description and results of this study have been presented in an article entitled, “The Current Status of Health and Social Services for Autism in Iran: From Parents’ Perspectives”. In sum, interviewees included 10 parents (mothers) of autistic patients who were living in Tehran, Iran, and nearby towns. These parents were selected from one private child psychiatric clinic and one NGO located in Tehran. The interviews covered areas such as initial signs of patients and worries of parents, contact with specialist, diagnostic procedures, using special training centers and training courses, and burden on the family (18).

***Study three***

A qualitative study was done using a semi-structured interview with a sample of service providers in the field of autism in Iran.

The service providers were two psychiatrists (a child psychiatrist and a general psychiatrist who, in addition to their practice as psychiatrists, have been in charge of positions in the mental health care system of Iran and mental health research centers for a long time), four psychologists, two occupational therapists, and two speech and language pathologists. The interviews were conducted in their offices and lasted about 60 minutes on average. All the interviews were voice recorded and transcribed by one of the investigators.

The interviews consisted of questions on general information about participants’ educational background and history of practice, autism relevant courses they passed, rate and age of the clients with autism, process of evaluation and diagnosis, current services and interventions in the community, causes of autism, barriers to use and access the services, current supports for people with autism and their families, services and supports required for people with autism, their personal practice and its barriers, and their personal experience of working with people with autism and their families. 

**Step two**

Identifying current evidence-based services

To achieve this goal, a systematic search was conducted to find reviews, systematic reviews, and meta-analyses which studied evidence-based services for autism. Also, two English databases (PubMed and Scopus) were searched from 1979 up to 2015 and the obtained articles were screened based on their title and abstract to find relevant articles. Articles on pharmacological therapy and adjunctive therapies, such as art therapy and studies that used animals, were excluded.

**Step three**

Designing the first draft of the package and its core elements

The data collected in previous steps were discussed in the expert panel, and core elements of the service package were determined based on local characteristics, needs and demands, and services and interventions that have proved to be effective. To choose the best services and interventions, some priorities such as feasibility, costs, effect size, cost-effectiveness, equitability, coordination with current programs, and services in the country were considered. This was done by comparing intervention models based on the quantitative data obtained from two previous steps and experiences and opinions of the expert panel (Table 2).

## Results

Since each step could be considered as an independent study, the results of each step were presented separately.

**Step one**

Step one consisted of three studies. The first study has been published (17) and the second and third studies are under review as separate papers. We will briefly present the results of these three studies that are applicable to the final goal of the project.

***Study one***

A total of 206 papers published during 1979 and 2015 in peer-reviewed journals by Iranian researchers were retrieved. Among them, 95 papers were published in Persian and 111 in English language journals. The number of articles has increased in recent years. In terms of subject area, studies on non-pharmacological intervention, and biological studies were the most prevalent, with 64 and 61 papers, respectively. Important topics, such as services, life span issues, and infrastructures have not been adequately studied. Based on findings of this study, no screening program exists for toddlers who are at risk of autism in Iran, nor does any evaluative information exist on the current services (17). This indicates that there is not enough evidence for providing services in the field of autism in Iran and the present study can be considered as a pioneer in this topic.

***Studies two & three***

The Main Findings of the Family Study

The mean age of children was 91.7 months (age range: 40-173 months) and the respondents’ (mothers) mean age was 36.3 years (age range: 24-46 years). The first clinicians that parents had contacted were pediatricians, internists, or speech and language pathologists. However, most of the children had been diagnosed by a child psychiatrist. Occupational therapy, speech therapy, and applied behavioral analysis (ABA), at clinic and home, were the most common services that families had used. Although high cost and limited availability were the most important barriers to service use, families preferred using services provided by private centers. There is no adequate financial support from the government and rehabilitation and educational services are costs were not covered by public insurance. However, parents need to be educated about their child’s condition, and children also need high quality services and appropriate residential centers (18)

The Main Findings of the Service Providers Study

Based on the responses received from service providers, there is no specific topic in university curricula about the diagnosis of autism and education of autistic patients. Service providers receive some training in their internship period. Child psychiatrists, as expected, are the sole group of service providers who receive training about diagnosis of autism and its pharmacological management.

The process of evaluation and diagnosis is not usually based on predefined protocols and assessment tools, but it is usually based on the DSM criteria. The age of diagnosis has been reduced compared to the past, but it is still not diagnosed on time.

Some supports from the Social Welfare Organization and charities are available for people with autism, but high costs and shortage of eligible specialists are serious problems. 

Inadequate evidence-based material and training courses and lack of standardized assessment tools for patient evaluation and intervention plan development are the most important obstacles that service providers face in their routine practice.

**Step two**

Scopus and PubMed databases were searched from 1979 up to 2015 using relevant keywords; the search was limited to reviews and meta-analyses. After removing duplicates, 965 articles remained and were screened based on title, abstract, and full text. In addition, Google scholar was searched to find other relevant articles.

General Guidelines for Providing Service 

Given what was needed for step three, the WHO meeting report on autism and other developmental disorders (19) was found to be most useful and practical. In this report, the points that should be considered by stakeholders for providing appropriate and effective services have been recommended. In brief, the key recommendations of this report are as follow:

- Diversity in abilities and needs of people with autism should be considered.
- There are evidence-based psychological interventions that are effective in reducing core symptoms and increasing adaptive behaviors; however, they need much resources to operate. The evidence for accessible and feasible service delivery models and effective strategies is needed to strengthen the infrastructures and resources.
- Involving parents and paraprofessionals can significantly increase access to interventions.
- Resource allocation should be based on the local needs assessment.
- Primary health care system has a good setting and infrastructure to play an important role in early screening, providing care, and care coordination.
- The collaborative models of care and task sharing approaches using multidisciplinary teams from the community are recommended.
- Increasing human resources is critical.
- Evidence-based materials should be provided for different types of service providers in each country.
- Early screening for autism should be included in current child health services.

The WHO report, as a road map, has emphasized on early screening, early interventions, and providing the required infrastructures based on local priorities and characteristics. 

***Early Detection***

There are some reviews about early screening of autism. In a comprehensive systematic review, Daniel et al have investigated methods of early identification for children who are at risk of autism (20). They reviewed 40 articles that had used 35 approaches to early identification. Those approaches were summarized in three categories: 1) four articles were about increasing public awareness regarding early development and autism symptoms; 2) 21 articles focused on routine screening, and 3) 10 articles were based on practice improvement. In sum, routine screening was the most effective approach. Other findings of this systematic review were as follow: early screening programs are conducted in primary care settings; 18-24 months old children are recommended to be visited for early screening of autism; and multilevel screening programs using short parent report questionnaires as the first level and more rigorous evaluations by diagnostic tools or trained specialists in the next levels are feasible and effective for early detection of autism.

***Early Intervention***

More evidence is available on early interventions. Overall, intervention methods can be divided into 2 broad categories: 1) comprehensive intervention models that cover all areas affected by autism, and 2) specific intervention models that focus on specific areas, such as communication.

Odom et al (21) systematically reviewed the literature about comprehensive intervention models in the field of autism to provide evaluative information for service providers and families to make decisions for adaptation and/or selection of an appropriate model. The comprehensive model is defined as an intervention that covers all affected developmental areas in autism, with a duration of more than one year and more than 25 hours per week. In sum, they found 30 comprehensive models, most of which were based on behavioral principles. They classified these models in five categories: 1) applied behavior analysis—clinic or home based; 2) applied behavior analysis—classroom based; 3) applied behavior analysis—inclusive; 4) developmental and relationship-based; and 5) idiosyncratic. They have also evaluated these models based on six dimensions: operationalization, implementation measures, replication, type of empirical evidence, quality of the research methodology, and complementary evidence from studies of focused interventions. In each dimension, a model can take a score of zero to five. Based on the definition, the models that have received the scores of four or five in at least four evaluated dimensions have stronger evidence. These models were as follow: the Denver model (developmental- and relationship-based); the Lovaas Institute (applied behavior analysis—clinic- or home-based); the May Institute (applied behavior analysis—classroom-based); the Princeton Child Development Institute (applied behavior analysis—classroom-based); and the LEAP (applied behavior analysis—inclusive). For more information, you can refer to original works (21).

There are other models that have lower scores but are still appropriate: Autism Partnerships (applied behavior analysis—clinic- or home-based); CARD (applied behavior analysis—clinic- or home-based); Children’s Toddler Program (applied behavior analysis—inclusive); DIR (developmental- and relationship-based); Douglass (applied behavior analysis—classroom-based); PRT (applied behavior analysis—clinic- or home-based); Responsive Teaching (developmental- and relationship-based); SCERTS (developmental- and relationship-based); and TEACCH (idiosyncratic) (21).

There are some other reviews that can be informative for the expert panel’s decision-making. In a meta-analysis, the studies that had compared early behavioral interventions with treatments as usual or no treatments were investigated. This meta-analysis included 1 RCT and 4 clinical trials, with a total sample of 203 children younger than 6 years. This study has shown that early intensive behavioral interventions have a positive effect on different developmental domains (effect sizes g = 0.69 for adaptive behaviors, g = 0.5 for expressive and receptive language, g = 0.42 for socialization, g = 0.55 for daily living skills, g = 0.74 for daily communication skills (22).

A systematic review was done on the studies that have examined the efficacy of the early start Denver model (ESDM) during 2010 and 2015 (23). The refined version of the original Denver model is ESDM that combined principles of behavioral and developmental approaches to be more appropriate for children younger than 12 months. This review included two randomized controlled trials, four controlled trials, and two observational cohort studies. This review revealed that ESDM is an effective intervention for very early childhood years and increases cognitive, language, and adaptive skills. It can also be efficaciously delivered by parents and in community group settings.

Moreover, the internet and some handbooks were searched for more information about comprehensive models, including ages served, areas of intervention, delivery model/s, intensity and duration, delivery setting/s, parents’ role, materials (manual, curriculum, test, etc.), and the required expertise and other information. This information was used in addition to other evidences for making decisions in the expert panel.

In sum, the obtained information emphasized early detection and early intervention. As the best practice, High-risk toddlers should be evaluated by reliable diagnostic tools or eligible specialists for making final decisions about the diagnosis. After detecting suspected cases, they should be enrolled in appropriate early intervention programs. Early intervention models based on the principles of ABA have most evidence, however, for younger children, it should be combined with principles of developmental and more naturalistic approaches. Using parents and paraprofessionals has also been recommended to increase intensity and accessibility of intervention.

**Step three**

All information obtained from previous steps was discussed in the expert panel to make decisions about an appropriate service package to meet the needs of children with autism and their families in Iran.

Based on the available evidence from previous steps, the members of the expert panel concluded that a comprehensive service package should consider these issues: 1) early detection as the first step in service delivery; 2) supporting families to use current services conveniently and appropriately; 3) training people with different levels of expertise with evidence-based material to overcome the shortness of human recourses; 4) designing and adapting evidence-based interventions in the form of specific and predefined protocols, manuals, and materials that can be taught and used with high fidelity; 5) using reliable assessment tools in the process of child evaluation, interventional goal setting, and modification; 6) using an intervention model with a multidisciplinary approach that can be used in conjunction with community-based service delivery models; and 7) focusing on parents as a critical part of intervention teams.

The experts suggested that a service package with 4 core components to respond to perceived needs in Iran should be designed and implemented. The components are as follow: 1) early identification of at-risk children; 2) care coordination and facilitation of access to current services; 3) implementation of an evidence-based early intervention program; and 4) training interventionists using an effective educational framework, based on evidence-based materials.

A brief description has been presented for each component as follows:

1) Early identification of at-risk children

A three-level screening program is recommended. As the first level, children who are brought to health centers for 18-24-month vaccinations will be screened using a brief parent report questionnaire. The recommended questionnaire is the short version of Quantitative Checklist for Autism in Toddlers (Q-CHAT) (24). The Q-CHAT is the extended version of CHAT and covers broader symptoms of autism. The definition of autism has changed from a categorical grouping to a dimensional category in the last edition of DSM. Thus, it is highly recommended that autism be screened by the tools that consider autism as a continuous concept. Also, the Q-CHAT is suggested to be an appropriate tool for this purpose (25, 26). Initial validation study of the Persian version of this questionnaire has been already conducted (27). All screening positive cases will be sent for further evaluation to the second level of screening. In level two, parents will be interviewed over the phone based on the toddler algorithm of Autism Diagnostic Interview-Revised (ADI-R) (28, 29). Telephone administration is recommended because previous studies have shown that a minority of parents did not come for further evaluation (29). As the third level, children who are suspected to have autism based on the telephone interview will be sent for final evaluation based on the DSM-5 diagnostic criteria to a hospital, clinic, or a community mental health center (CMHC) with pediatric services. In each referral center, there should be at least one child psychiatrist, or general psychiatrist, or child neurologist, or pediatrician, who should be trained and eligible for diagnosis of autism in young children and toddlers. The final decision about diagnosis is based on the evaluation by one of the above-mentioned eligible specialists.

All these procedures will be facilitated by a case manager to increase the number of cases that completed all three levels.

2) Care coordination and facilitating access to current services

Care coordination can help families to access relevant appropriate care more easily. In this regard, establishing a registry of service providers in the field of autism is recommended. All the licensed service providers can register in this registry, based on their expertise and catchment area. Service users can directly use the registered data or contact a care coordinator who is part of this system to choose appropriate services.

3) Implementing an evidence-based early intervention program

In sum, a comprehensive intervention program that can be used for very young children (18- 24 months) and includes parents should be delivered by different providers and in different settings (team-based) using a multidisciplinary approach. Based on the comparison between characteristics of the existing early intervention models that are supported by evidence, our recommended program should include principles of ESDM as the main approach for early intervention (Table 2). Given that there might be some cases who need a more adult directed approach or visual strategy, we will also include principles of ABA, TEACCH, and PECS in our intervention program. 

4) Training interventionists

To overcome one of the main determinants of disparities in access to care, it is critical to train an adequate number of interventionists using evidence-based models. Defining and implementing training courses in different levels is the fourth component of our service package.

Details of the procedures and the required materials for each component have been prepared and are ready for pilot testing.

## Discussion

Nowadays, autism has become an important challenge for the health care systems, and for all communities around the world. Early identification and intervention are the only solution to reduce burden of ASD (1). Iran is not exempt from this problem, and thus extending current services for autism based on evidence-based and cost-effective models is critical for the health care system of Iran. The most important gap in adopting intervention models is that focus is mostly on the efficacy data that belong to academic settings, and typically these models do not work appropriately in real community settings. Thus, we went through REP framework, which is recommended by the CDC, that can guarantee the effectiveness of intervention packages in real intervention settings in communities. This framework has four phases: 1) preconditions, 2) preimplementation, 3) implementation, and 4) maintenance and evaluation. We have completed the first phase of this framework and developed the first draft of a comprehensive services package based on the local needs and the characteristics of the PHC system in Iran. The package consists of four complementary components: 1) early identification of at-risk children, 2) care coordination and facilitation of access to current services, 3) implementation of an evidence-based early intervention program, and 4) training interventionists using an effective educational framework using evidence-based material. The next 2 phases of the REP framework, which are recommended for the early stage of service adaptation, should be completed before the final format of the package could is considered for dissemination. Thus, another study is needed to investigate the pre-implementation and implementation phases of the REP framework of our package. 

The REP framework was originally developed to implement HIV interventions in community-based settings. It was successful in its primary field and has recently been used in other fields, such as packaging mental health services (14, 30). In a recent study, the REP framework was used to adopt a psychological intervention for caregivers of children with nodding syndrome in the public health system of Uganda (15). Following the 4 steps of REP framework led to a successful service implementation; they chose group interpersonal therapy (IPT-G) (in preconditions step) and effectively delivered it to more than 90% of beneficiaries.

Our proposed service delivery package has several potential advantages. It includes an early detection component based on the current evidence, which is crucial to increase the efficacy of early intervention and later outcomes (31). It is shown that early detection of at-risk children can reduce the burden of autism during life time (8). Our detection system considers new changes in the concept of autism (using Q-CHAT as a dimensional screening tool) and feasibility of implementation of such a system. Also, we proposed health centers as a setting of global screening that maximize its coverage, while it does not take much recourses and uses telephone interview in the second level to increase the rate of participation of families.

The care coordination component of our system can also help families to access appropriate services. There are some resources in the society, but families usually are not aware of or cannot make a decision about best available services in their residency area. Thus, many families postpone the intervention and feel confused and lonely. The registering service providers and receiving help from a care coordinator can increase access to available services and accelerate intervention.

Appropriate services should be provided after any screening program, and cases should have easy access to the services. An efficacious early intervention for toddlers with autism should consider some issues, such as combining behavioral and developmental approaches, susceptibility to delivery in different naturalistic settings, covering different affected areas in autism, comprehensiveness, intensity, and parent involvement (32). In this study, we compared available comprehensive intervention models and selected principles of ESDM as the main body of our intervention model. ESDM have several advantages for younger children with autism compared to other models, such as combining principles of developmental and behavioral approaches, using multidisciplinary teams, parent involvement, and delivery in different settings by different practitioners (Table 2). Given the diversity among learning styles and needs of toddlers with autism, we also included principles of ABA, TEACCH, and PECS in our intervention model.

**Table 1:** Outline of REP Process for Health Services-Based Interventions and Their Counterparts in the in a Low Resource Setting

**REP process**		**Counterparts**
**Phase**	**Activity & Process**	**Activity & Process**
Preconditions	Identify need --Identify high-burden condition --Identify barriers to implementation Identify barriers --Organizational needs assessment, usual care	**Step one**: Need assessment and situation analysis **Step one-study one**: A systematic review on the studies in the field of autism by Iranian researchers on local samples **Step one-study two**: A qualitative study using semi-structured interview on a sample of parents of people with autism **Step one-study three**: A qualitative study using semi-structured interview on a sample of service providers in the field of autism in Iran
	Identify effective intervention --Identify intervention tested in a completed, randomized controlled study	**Step two**: Identify current evidence-based services A systematic review by relevant keywords to -- find reviews, systematic reviews and meta-analysis which tested evidence-based services for people with autism --- find reports and recommendation about service providing
	Draft package --Write package into everyday language --Distinguish core elements, menu options	**Step three**: Designing first draft of the package and its core elements At this step, the data retrieved from the previous steps were discussed in the expert panel and core elements of the service package were determined based on the local characteristics, needs, and priorities and effective services and interventions by considering some priorities such as feasibility, costs, effect size, cost-effectiveness, equity, coordination with current programs and services in the country.
Pre-implementation		
Implementation		
Maintenance and evolution		

REP: Replicating Effective Programs

**Table 2:** Comparing Different Intervention Programs Based on Their Characteristics Assumed to be Necessary for the Low Resource Setting

	**ABA**	**ESDM**	**PECS**	**TEACCH**
Combining developmental and behavioral approaches		**		
Multidisciplinary approach		**		*
Appropriateness for younger ages	*	**	*	*
Comprehensiveness	**	**		
Parent involvement	*	**	*	*
It can be widely used in different settings and by different peoples	*	**	*	
Specific assessment tool for evaluation of developmental level and progress		**		*
Specific assessment tool for intervention plan development	*	**		*
Manual and predefined protocol	*	**	**	*
Using visual strategies			**	**
Environmental modification				**
Focus on severe behavioral problems	**	*		

ABA: Applied Behavior Analysis

ESDM: Early Start Denver Model

PECS: Picture Exchange Communication System

TEACCH: Treatment and Education of Autistic and Related Communication Handicapped Children

## Limitation

The most notable limitation of the present study was time construction. Also, the package was not pilot tested; however, it will be done in the next proposed study. The other limitation of the study was that the sample size of qualitative studies was relatively small.

## Conclusion

REP framework was used in the present study, which has shown to be effective in adaption and implementation of health care services. After reviewing the preconditions of REP, we drafted a comprehensive services package with 4 components for toddlers with autism in Iran. As both clinical effectiveness and implementation of the package needed to be evaluated, a multicenter hybrid effectiveness-implementation randomized control trial should be conducted for the next step (33).
